# Towards Dialogic Metaphors of Learning – from Socialization to Authoring

**DOI:** 10.1007/s12124-021-09661-5

**Published:** 2021-10-07

**Authors:** Tina Kullenberg, Roger Säljö

**Affiliations:** 1 Department of Educational Science, Specializing in Primary and Secondary School, and Special Needs Education, Kristianstad University, SE-291 88, Kristianstad, Sweden; 2 Department of Education, Communication and Learning, University of Gothenburg, 40530 Göteborg, Sweden

**Keywords:** Learning, Bakhtin, Socialization, Polyphony, Authorial learning

## Abstract

The background of the article is an interest in theories of learning and the metaphors of learning they build on and propagate. The basic argument is that the discursive construction of learning plays a central role in theoretical perspectives in research but also in discussions of societal issues in a wider sense. An initial observation is that current metaphors of learning oscillate between emphasizing socializing/reproductive dimensions and perspectives which foreground new-thinking transformations of existing collective knowledge; the culturally given. Hence, our aim is to explore conceptions of learning underpinning dominant theoretical perspectives as behaviorism, cognitivism, pragmatism, and various sociocultural traditions, in the light of this theoretical tension. Our conclusion is that the views of communication and learning inherent to the radical dialogic perspective on communication that stresses the unfinalizable nature of knowing, offered by Bakhtin, add to our understanding of how learning may be conceptualized in contemporary society. Such a dialogic perspective, emphasizing open-ended agency, plurality of voices, and performative potentials of creatively expressing opinions when learning from each other, offers a perspective on learning worth considering in times of diversity, unpredictable risks, and the need for critical self-reflexivity.

## Introduction

The concept of learning plays an important role in discussions about individuals, social institutions, and society more generally (Säljö, 2015). In societies characterized by innovation, global competition and rapidly changing technologies and social infrastructures, learning is often seen as the mechanism by means of which the population and the labour force will be able to keep up with, and contribute to, societal development. Expressions such as life-long, life-wide, and work-place learning have been launched in recent decades to emphasize the role of learning throughout our lives (European Commission, 2007; Jarvis, 2010). The rhetorical power of the term is obvious, and learning is almost always used with positive connotations; the more people learn, the better.

Given this open and suggestive character of the concept and its widespread use, it comes as no surprise that it has been very difficult in research to reach an agreement on exactly how to define learning. Most introductory volumes point this out (cf., for instance, Borger & Seaborne, 1976, p. 10ff; Hilgard & Bower, 1966, p. 2ff; Murphy & Knight, 2016, p. 404ff). It is repeatedly argued that learning is a central feature of human life, and yet it is difficult to pinpoint exactly what is shared between all those instances that we may meaningfully talk about as learning. The dilemma that follows, some argue, is that if we cannot define a concept in a reasonably coherent and exhaustive manner, how can we then ever hope to explain it or use it as an element of credible explanations? However, this may be considered as a welcome opportunity rather than a problem. Conflicts about definitions of concepts often signal that there are interesting tensions of a paradigmatic nature in research perspectives that should be drawn out into the open and discussed. In the following, our point of departure is that theoretical conceptualizations of learning cannot, and should not, be understood as pointing to a single and clearly delimited phenomenon that has an independent existence. There is no “it” that can be unequivocally defined.

Accordingly, this article will not attempt to formulate intractable, finalized claims about how to define learning. Rather, we attempt to explore what dialogical metaphors of learning may offer as a contrast to conventional conceptualizations in the field. We, thus, focus on problematizing traditional and individualistic views on learning in our attempt to sketch the contours of conceptions of learning that would seek to follow a more radical dialogic epistemology inspired by Bakhtin and educational scholars who have taken his dialogue philosophy further in different directions. We do this by exploring the metaphors inherent to some of the dominant learning theories, arguing that metaphors play an important role in this context. The overall ambition is to contribute to a discussion between various approaches to knowledge, learning and development in contemporary society. Besides the initial considerations regarding what is indicative of learning and developing in a late modern way of existence, we go on to explore what kinds of theoretical conceptualizations might be relevant and how Bakhtin’s dialogism may contribute to elucidate conceptual facets of knowledge that are not to be overlooked at present.

The text is organized as follows: First, to set the scene, and without attempting to be exhaustive, we attend to some central features of a late modern framework for individual learning and development. Following this, we review existing learning metaphors representative for distinct traditional research perspectives. Next, we turn to Mikhail Bakhtin to explore some elements of his philosophical perspectives on communication, in particular his ontological claims about the fundamental openness, creativity and unfinalizability of communicative encounters, and our capacities to learn from each other. We also consider the epistemological implications for understanding knowing and learning, following such a perspective, by discussing the role of dialogic metaphors within the field of pedagogy and contemporary society.

## Living and Learning in an Age of Pluralism and Risks

In his well-known book *Risk society: towards a new modernity* (1992), the sociologist Ulrich Beck addresses the meaning of societal *risk* in a double sense. At the time of its publication, environmental concerns were receiving increasing attention as part of the emphasis on risk, but on the theoretical level his ambition was to point to the question of the unpredictability and risk-loaden nature of many activities in late modernity. His colleague, the sociologist Anthony Giddens (1990), conceptualizes this specific era of modernity as “radical modernity”. Giddens also discusses the emerging new social order where individuals psychologically must handle a range of experiences of unforeseen risks and danger, in contrast to the era of security and trust (p. 7). He points to the fact that “the nature of modern institutions is deeply bound up with the mechanism of trust in abstract systems, especially trust in expert systems” (p. 83). This implies that individuals in radical modernity have to cope with existential anxieties when they are forced to give up established features of their basic sense of “ontological security” (p. 92), as societies transform into social orders that rather accentuate the need of living with rapid, unforeseen changes as existential premises. Giddens prefers the notion of ontological security to the unconscious, emotional state of being-in-the world used by some philosophers to characterize continuities of identities and practices. The constancy of surrounding social and material environments contributed to the sense of stability and reliability in life.

Turning to the late modern conditions of young people, the childhood sociologists James et al. (1998) already two decades ago argued for a radical shift in how researchers should speak about young individuals and their life careers. One of their decisive objections was the rhetoric of singularity when analyzing childhood(s) (p. 125f.). In their seminal book *Theorizing Childhood*, the authors claim that it is necessary to pay attention to subjectivity and agency of children. Accordingly, they proposed the idea of children as legitimate (social) *actors* and *citizens* in their own right as an alternative to considering them as being in a state of becoming. The latter view implies a vision of postponed agency rather than one that is realized in the here-and-now. The authors argue that this traditional line of reasoning belongs to “transitional theorizing about the child” and “the process of this inculcation is referred to as socialization” (ibid., p. 22). In our opinion, the authors correctly problematize a reductionist perspective on children’s being, a view where their current life experiences, competencies, opinions etc. are not acknowledged as legitimate in their own right for engaging in societal practices. Thus, the view on childhood traditionally has been premised on a specific kind of instrumentality, where the future role as an adult should provide guidance for socialization.

In a similar vein, scholars in education have pointed to the need of rethinking children and childhood in the context of institutional learning and development. For example, Veraksa and Sheridan (2018) recognize the instructional consequences of preschool children’s agentic subjectivity, and they argue for deliberately involving young students in decision-making and having a say in designing their learning experiences (cf. Kullenberg, 2019). Likewise, the notion of children’s voices has been addressed in a wealth of studies in education to point to the changing nature of participation in instructional practices. However, as Komulainen (2007) notes, this debate cannot be reduced merely to concern schooling and children’s right of being heard by teachers and other adults. Issues of children’s agency in society go far beyond this.

Recently, scholars in several academic disciplines have discovered the value of Bakhtin’s contributions for understanding communication and human agency in current, pluralistic and rapidly transforming societies. Also, scholars in the field of educational science have started to pay attention to his perspectives as a platform for understanding learning and the communication and sharing of experiences. Even though we must keep in mind that Bakhtin was not a theorist of learning, he was an influential and engaged educator who has written about the field of education (Bakhtin, 2004; Bazerman, 2005). His writings on the architectures and dynamics of communication have important implications for our understanding of epistemological concerns as learning and teaching (Matusov, 2007; Rule, 2015, p. 37; White, 2016).

Furthermore, his dialogue philosophy has much to offer when considering what learning implies in contemporary society (Brandist et al., 2017). As Teo (2019) points out, Bakhtin-influenced analyses of education and learning address significant forms of knowledge in an increasingly globalized world. Here, learners are encouraged to develop what is often referred to as twenty-first century skills that go beyond the reproduction of ready-made knowledge, such as innovative creativity, critical thinking, self-reflexivity (Beck et al., 1994) and the capacity to engage in complex forms of being and communicating characterizing pluralistic societies. Consequently, current societal circumstances warrant a complementary research focus that seeks to understand learning and identity formation based on premises relevant for complexity and diversity.

## Metaphors of Learning: A Brief Review

Twenty years ago, Sfard (1998) published her much-cited article on two contrasting metaphors of learning. The particular distinction that Sfard focused on was that between perspectives that conceive of learning in terms of an “acquisition metaphor” and those that build on a metaphor of “participation.“ The former metaphor—acquisition—characterizes cognitively orientated perspectives on human thinking “and makes us think about the human mind as a container to be filled with certain materials and about the learner as becoming an owner of these materials” (Sfard, 1998, p. 6). This metaphor, in turn, is rooted in the “conduit metaphor” of communication, described by Reddy (1979). Here, communication is construed as the transmission of information from a speaker (sender) to a listener (receiver), and, if successful, this transmission results in the listener, following Sfard, “owning” the same information and/or concept as was sent by the sender. The technical metaphor of transmission that underpins this model is obvious. In contrast, the participation metaphor construes learning as emerging through involvement in communities, where individuals increase their capacities to participate in, and contribute to, collective practices by moving from the periphery to the centre of the activities that sustain communities. This participation metaphor is grounded in an anthropologically inspired, explicitly anti-cognitivist, view of knowing and knowledge reproduction as situated in social practices, articulated by Jean Lave and others (Chaiklin & Lave, 1993; Lave & Wenger, 1991). In this approach, the cognitive level is not privileged as the object of inquiry; rather, the situated appropriation of material, social and intellectual constituents of activities is at the centre of attention as are the identity shifts involved when moving from the periphery to the centre of a community of practice. An important element of Sfard’s argumentation is that these two metaphors are not theoretical in a precise sense. Rather, they lie behind and underpin much of the theorizing that goes on in the behavioural (and other) sciences; they are, in a sense, pre-theoretical, and, we might add, constitutive of how learning is understood.

From a traditional scientific point of view, the conclusion that the concept learning is metaphorical may be seen as making it irrelevant for theorizing. However, if we follow the perspective articulated by Lakoff and Johnson (1980) of metaphors as something “we live by”, we may turn to the functions that metaphors play in everyday life, in scientific contexts, in policies and in institutional settings including politics (Brown, 2003). For instance, in psychology the metaphor of the mind as a computer played a significant role in triggering research on human “information processing”, “memory systems” and “perceptual mechanisms” and the role these components play in the “acquisition of knowledge” (cf., for instance, Hunt, 1971, who in a leading psychological journal explicitly asked "what kind of computer is man?"). Indeed, this process metaphor shaped most of what we now know as cognitive psychology (cf. Bruner, 1990, for a critique of the human information processing metaphor in psychology, which, in his view, focuses on computation rather than meaning-making).

An interesting, and very important, feature of metaphors is that in many situations “we are unaware of the metaphors that shape our perception and understanding of social situations” as Schön (1993, p. 266) points out. For instance, and returning to the metaphors of cognitive psychology, when speaking of memory as a set of processes operating in mechanical systems (short-term memory, long-term memory, working memory etc.), it is natural to say that what we do when we remember is that we “store” memories and “search” our memory systems in order to “retrieve” information. This “things-ontology” (Shotter, 1993) converts the complex, constructive, tool-dependent and generally collaborative activity of remembering into a mechanical search of a static body of information already stored in a “system” (cf. Kullenberg, 2019; Mäkitalo et al., 2017; Säljö, 2002).

In this perspective, analysing theories of learning in terms of the metaphors of learning they suggest and cultivate is thus not just a conceptual and scholarly enterprise of interest to specialists. Instead, metaphors of learning play an important role in society, and the metaphors adopted co-determine decisions that regulate curricula, access to education for various groups and the evaluation of outcomes of education. Even though there are sharp conflicts between various theoretical traditions, for instance between behaviourists and cognitivists, a shared element is that learning is a matter of reproduction of what is already known. For the behaviourist, the Stimulus–Response connections of conditioning imply copying behaviours that can be defined ahead of time. In cognitively orientated traditions, focusing on the acquisition of conceptual knowledge, learning is successful to the extent the learner is able to reproduce and handle a given concept according to some predefined, usually scientific, standard. Furthermore, in both these instances, the assumption is that when the goal of successful reproduction has been reached, learning, as it were, is complete.

This construction of learning as basically limited to reproducing what is already known has a long history in society, in educational practices and in the wider interpretation of what learning is all about. And, of course, there is some substance to this line of thinking; societies rely on processes that make their “cultural memory” (Donald, 2018) accessible to new generations. But, the problem is if it is reasonable to assume that these, essentially reproductive metaphors, tell the full, or even most interesting, story of what it means to learn.

Over the centuries, alternatives to this reproductive metaphor have been suggested. One well-known example of such an alternative is Dewey’s concept of inquiry and the idea of transactional perspectives on actions/activities, as suggested within the pragmatist tradition (Dewey & Bentley, 1949; cf. Clancey, 2011). He explicitly objected to a view of learning which rests on a metaphor of “pouring knowledge into a mental and moral hole which awaits filling” (Dewey, 1966, p. 51). To support “growth” (ibid. p 41), there is a need to recognize that the end is not predetermined. In this perspective, learning is understood as a constitutive element of an ever-developing organism-environment relationship. To characterize the dynamics of such relationships, Dewey makes a distinction between inter-action and transaction that refers to the ways in which the organism-environment connection evolves. Inter-action implies that the interacting elements stay the same during the activity, while transaction implies that the elements of an action are reorganized to form a new organism-environment relationship. A key element of this dynamic relationship in the context of learning is inquiry, which is conceived as “the controlled or directed transformation of an indeterminate situation … into a new “unified whole”” (Dewey, 1938, p. 108). Thus, inquiry “emphasizes that learning is an active, dynamic process of investigating, probing reformulating, hypothesizing, examining, manipulating, deducing, theorizing, experimenting” (Clancey, 2011, p. 250). Adopting Dewey’s point of view, learning cannot be separated from the situation and the actions that evolve, nor can it be divided into separate subcomponents or subprocesses that causally explain an outcome. Rather, learning is an integrated feature of an activity, and it incorporates a range of actions. This is clearly an alternative conceptualization of learning.

## Sociocultural Conceptions: Tools and Voices

Vygotsky’s contribution to theorizing learning and development needs no further introduction today. What makes it important to recognize his work in the context of our contemporary considerations of what it means to learn is that he, among his many contributions, paved the way for a new and interactionally oriented psychology, nowadays often referred to as a sociocultural or cultural-historical perspective on learning and development. In his effort to link thinking, as a mental activity, to interactional and broader social and historical dimensions of cultural practices, Vygotsky (1978, 1981) put learning and development at the centre of our understanding of psychological functions. These functions are essentially tool-dependent, that is contingent on human conceptual and material innovations and cultural-historical contexts that develop over time. Furthermore, they continuously undergo change. In this sense, “learning is a necessary and universal aspect of the process of developing culturally organized, specifically human psychological functions" (Vygotsky, 1978, p. 90).

Several scholars have extended Vygotsky’s interpretation of the development of psychological functions to include dialogical perspectives on communication. Rogoff (1990) and Wertsch (1998) both discuss, and reinterpret, the Vygotskian notion of internalization in the context of Bakhtin’s (1986, 1990) analyses of communication. Both authors point out that, in the Vygotskian conception of human psychological functions as emerging through “internalization” of “interpsychological categories” that become “intrapsychological categories”, there is a risk that the concept of internalization is understood as “a kind of opposition, between external and internal processes that all too easily leads to the kind of mind–body dualism that has plagued philosophy and psychology for centuries” (Wertsch, 1998, p. 48). Rogoff (1990), analogously, points out that internalization is often interpreted too literally as if it were about something passing a barrier between the external world and the mind. To avoid these unfortunate connotations, Rogoff, Wertsch and others have suggested that the alternative, Bakhtin-inspired, concept of appropriation should be considered (Wertsch, 1998, p. 53ff.), since it represents a more viable and dynamic interpretation of the ways in which such processes occur. In the famous formulation by Bakhtin, the “word in language is half someone else’s. It becomes “one’s own” only when the speaker populates it with his own intention, his own accent, when he appropriates the word adapting it to own semantic and expressive intention” (Bakhtin, 1981, p. 293), an alternative metaphor of learning becomes visible.

One of the most famous quotes from Vygotsky is his claim that language is “the tool of tools.” However, he did not conceive of language as a static and fixed tool. His analyses of the tensions between “sense” and “meaning” testify to this (Vygotsky, 1987). Sense is always a local and situated accomplishment involving an agentic subject putting language (or other cultural tools) to use for particular purposes. Sense is also primary in the life-world of people, even the newborn child responds to and interacts with the world as a social being making sense of communicative initiatives of others (Kravtsova, 2017). Thus, for both Vygotsky and Bakhtin, language is not a neutral or ready-made medium. However, Bakthin emphasizes these dynamic features of language use even more clearly, as is evident in the claim that, a word or an expression is “populated-overpopulated-with the intentions of others.” Thus, “expropriating it, forcing it to submit to one’s own intentions and accents, is a difficult and complicated process” (Bakhtin, 1986, p. 294). Appropriation in the Bakhtinian sense thus emphasizes the dynamics of personal sense-making and, as such, it is part of an ongoing activity in which learners struggle with understanding the intentions and ideas of others beyond the intent of reproducing them. Thus, and this is crucial for Bakhtin and his followers, such a process of appropriation leads to people arriving at their own views, accents, intonations, and interpretations of utterances of speakers or writers. Their own ‘voices’ hence interanimate with others in multi-voiced discourses. We will come back to his dialogic interpretations of voices. To what extent this is practically viable or not in social life is an empirical question for him, since the cultural types of “speech genres” tend to prescribe what is possible to say and think (Bakhtin, 1986).

So, let us return briefly to the “ontological insecurity” characterizing late modern “risk society” (Beck, 1992) which calls for critical reflexivity and capacities for flexible attitudes of individuals. As a next step, we will seek to connect this discussion to the issue of learning and development in a dialogical and Bakhtinian perspective. We have singled out two salient and figurative concepts, which are especially fruitful for the purpose of contextualizing learning in a world that is no longer highly predictable, but rather dynamic, multi-cultural and pluralistic. What does it take to navigate and develop in such circumstances, and what could Bakhtin help us to notice? The two metaphors of interest here are *polyphony* (including the notion of “voice”) and *authorship*. As hinted, the overall idea of presenting these specific metaphors is to point to their potentials of reframing the idea of learning beyond the limits of the traditional emphasis on reproduction and socialization.

## Constituents of Dialogical Metaphors of Learning

### Voices in Polyphonic Encounters

The concept of voice plays a central role in Bakhtin’s (1999) analyses of dialogic meaning-making. As the dialogically minded linguist Linell (2009) points out: “utterances in talk are always carried by the individual voice” (p. 114), and they always include a “personal signature” (p. 114ff.). Linell continues to explicitly link this dimension of communication to the Bakhtinian metaphor: *voice as perspective on topics*:

This brings us to another, somewhat metaphorical but characteristically Bakhtinian sense of the term ‘voice’, namely, an expressed opinion, view or perspective, something that the person would typically say and presumably (at least at some level of intention) stand for. (2009, p. 116)

Translated into areas of institutional learning, neither teacher voices nor student voices are simply realized or attended to the moment speakers have a chance to engage in public talk in the classroom. Besides the fact that empirical research tells us that students of different ages often do not have many opportunities to speak out and engage in lengthy dialogues (see for example Hayes & Matusov, 2005; Nystrand et al., 1996), the invitation to express a student voice is not necessarily obvious in institutional settings. Segal and Lefstein (2016) comment on the notion of students’ voices as constituents of classroom interaction:

Whose voices are expressed and attended to in classroom discourse? And how do these voices play off of one another in creating new ideas and meanings? In particular, to what extent are students empowered to express their own voices, rather than reproducing the teacher or textbook’s authoritative discourse? Building on Bakhtin, Hymes and Blommaert, we argue that realizing voices involves (a) opportunity to speak, (b) expressing one’s own ideas, (c) on one’s own terms, and (d) being heeded by others. (2016, p. 1)

In multi-voiced discourses, where voices of all participants in an activity may be heard, respected and responded to, learning has a chance to occur in response to a multiplicity of ideas and values, sometimes confronting each other and sometimes existing in harmony. Those different voices also have the potential to clash with the learners’ mindset, or even his or her identity, encouraging the individual to further reflect on his or her personal stance. Bakhtin proposed a musical concept for such a multi-voiced discourse suitable for dialogic sense-making: the notion of *polyphony* (Bakhtin, 1999).

Bakhtin introduced this concept of polyphonic discourse by going beyond the common notion of harmonious or consensual communication as an ideal. He stressed the existence of alterity and conflictual tensions between personalized voices of individuals as constitutive elements of encounters. In addition, he recognized the critical tensions between ideologically impregnated discourses in practices, and the inherent links to the ongoing formation of individual consciousness. Hence, what is significant from Bakhtin’s point of view is the plurality of voices in friction: voices which sometimes confront each other, thereby creating unpredictable gaps between the interlocutors rather than consensual intersubjectivity. The latter concept of intersubjectivity in learning theory and didactics is often, though not always, limited to a definition that refers to shared and agreed mental understanding (cf. Matusov, 1996).

When commenting on its musical origin, the dialogue philosopher Dmitri Nikulin (2006, p. 46) argues that Bakthin’s (1999) use of of polyphony can be understood as follows.

[t]he appropriate musical metaphor that adequately represents the structure of interaction among a plurality of independent, yet not isolated, personal voices, voices which are capable of being uttered in each other's presence, is that of polyphony. A simple musicological definition of polyphony states that it is a texture in which two or more melodies or themes are played, or sung simultaneously by different voices, which enter the polyphonic texture at various moments in time.

In the context of musicians’ playing, Skidmore (2016) develops Bakhtin’s idea of polyphonic expressivity, noting that,

the idea of polyphony corresponds to the fact that a specific piece of music is often performed by many musicians playing (or singing) together as an ensemble, and that often they will playing different instruments – instruments with different ‘voices’, singing different melodies – at the same time to produce a combined effect, such as a jazz band, an orchestra or a choir. (p. 34)

While Skidmore refers to the musical origin of the term polyphony, Bakhtin (1999) uses it non-musically, that is, as a generalized metaphor for dialogic communication. Outside the musical context, there is another important implication, especially for instructional communication between teachers and students, related to issues of power in social relationships. The critical feature of a genuine polyphony is the plurality of unmerged voices and consciousnesses, i.e., fully recognized voices with equal rights to be heard and respected within dialogues. In this sense, Bakhtin clearly idealized democratic rights in human communication by emphasizing equal rights to be heard (cf. Hirschkop, 1999).

Translated to education, and contexts of teaching and learning, this suggests a critical perspective on the role of the dominant, maybe even authoritarian, voice emerging through, for instance, the voice of the teacher or the textbook (cf. Matusov, 2007). Although the teacher has a crucial function as a dialogue partner and facilitator, a polyphonic interpretation of teaching and learning highlights the teacher voice as *one* of many agentic voices simultaneously at play. Polyphony itself points to the centrality of an interplay of different participants’ expressed knowledge as an alternative to the established educational tradition in which the instructor’s voice should lead and generally not be questioned.

A polyphonic approach to knowing further makes space for the communication of ‘truths’ which are not yet finalized, and maybe never will be. That means, the polyphonic approach recognizes explorative, expressive knowledge-in-progress as an element of learning and instruction, rather than limiting such activities to the voices of ready-made truths:

The dialogic means of seeking truths is counterposed to *official* monologism, which pretends to *possess a ready-made truth,* and it is also counterposed to the naive self-confidence of those people who think that they know something, that is, who think that they possess certain truths. Truth is not born nor is it to be found inside the head of an individual person, it is born *between people* collectively searching for truth, in the process of their dialogic interaction. (Bakhtin, 1999, p. 110)

In these lines, Bakhtin explicitly recognizes both the dialogic and the open-ended nature of knowing and learning, not only with respect to ontology but also in terms of epistemology, i.e. how we come to know.

### Authorship

While exploring the dynamics of voices in polyphonic interaction pregnant with infinite potentials for learning and sharing of perspectives, we have discussed how the notion of truth is pertinent to the contemplation of a dialogical interpretation of learning. For Bakhtin and his analysis of Dostoevsky’s novelistic genre, “authorship” is a significant feature not only in the limited sense of referring to the author of a novel. On the contrary, also his literary characters represent authorial, equivalent voices in the polyphonic context of the ongoing dramas. Bakhtin points out that this “approach to characterization contrasts with that of authors such as Tolstoy, whose heroes are clearly subordinated to the monologic voice of an author speaking through and over them, rather than ‘alongside’ and ‘with’ them” (Rule, 2015, p. 38). Such “genuine polyphony of fully valid voices is in fact the chief characteristics of Dostoevsky’s novels”, Bakhtin (1999, p. 6) argues. Thus, every character has something important to tell, and every dialogic encounter between these literary heroes offers thought-provoking opportunities for (re-)thinking, for example, moral, ideological, and practical dilemmas and, accordingly, invites existential forms of learning. This is the transformative nature of multi-voiced tensions in a most dialogic sense.

Emerging from this Bakhtinian concept of authorship, the metaphor “authorial learning” has been suggested in the context of dialogic pedagogy (Matusov, 2011). As hinted at, authorial learning alludes to how students are expressing thoughts and opinions in communicative authorship. It hence represents the potential for creative and dialogic learning, as well as personal expressivity, yet within a multi-voiced context. Contrasting the dominant “technological approach” to education with authorial teaching and learning, Matusov highlights the role of the learners’ meaning-making, based on their own desires, interests, questions and initiatives beyond the alienation that institutional schooling tends to end up in. In the latter, “[m]any of the students leave the school based on the technological approach as’educational zombies’—they may perform well on tests and exams, but they are lifeless in the field of academics, as their "toolkit" acquired in school is alienated from them” (p. 23). By implication, authorial teaching offers the opposite: a kind of “performance art” based on the teacher’s and student’s joint authorship. He defines authorship as “the participant's bid for a unique creative contribution fully or partially recognized by a relevant community and by the participant him/herself” (p. 24). Of significance here is the conception of education as transformation of learners’ agency; the idea that such experiences allow for new forms of thinking and being that transcend pre-set givens (e.g., pre-given curricular goals, lesson plans, knowledge claims).

## Why Dialogic Conceptualizations of Learning?

Following the Bakhtinian perspective on the dynamics of interaction, and the dialogic perspective on the outcomes of such encounters, what consequences may be seen for our understanding of what learning implies? The assumptions of traditional perspectives on learning are steeped in a functionalist interpretation of society, where reproduction of “ready- made truths” is the core element. The challenge conventionally identified is that new generations should reproduce and adapt to what is already known as efficiently as possible. Mass education with clearly defined “learning outcomes”, to use EU-speak, illustrates a technology of accountability for living up to this ideal. The Bakhtin-inspired perspectives on how people share experiences have something important to contribute to societies which move from predictability to the types of dynamic and less stable societal configurations that Giddens (1990) and Beck (1992) point to. If we need to single out one essential insight from Bakhtin’s dialogism, it is his consistent emphasis on the value of appreciating and cultivating multi-voiced polyphony (that also has a democratic implication of value for our times).

As mentioned earlier, James et al. (1998), Veraksa and Sheridan (2018) and others argue that *transitional theorizing* of children’s development, where they are not ascribed agentic value in their here-and-now activities, has problematic consequences. Although we have seen a longstanding international trend of emphasizing so-called progressive and child-oriented teaching, there is still a lack of research and instructional practices of focusing on the children’s perspectives and voices as guidelines. Even in progressivist discourses the instrumentality of functionalist conceptions of what it means to learn and grow prevails.

Furthermore, one other thought-provoking element of a dialogic metaphor of learning is the suggestion to see learners/students as authors as Matusov (2011) has suggested through the concept of “authorial learning.” We have suggested the significance of individual and, implicitly, social creativity in terms of having recognized agency, and a voice, in learning. The notion of authorship—and authoring—contributes to a radical shift in positioning the learner’s agency. As Matusov (2011) underlines, it implies a radical kind of creativity as well. At the level of communication, such transforming creativity, and such outspoken agency as well, cannot not be realized under all institutionalized forms of interaction. It presupposes an environment that is open to knowledge contributions that go far beyond the conventional focus on reproduction of ready-made truths and authority patterns.

Developing environments that appreciate and promote authorship requires reconfigurations of patterns of communication. In institutional settings, this calls for some kind of awareness of educational design, although the design here does not imply imposing a unidimensional, normative definition of how events should unfold. Promoting authorship implies encouraging learners’ creativity to transcend the cultural given in terms of conventional rules, traditions, tools, ideas, and values, and the idea supports the assumptions of the unfinalizable character of knowing. In addition, the Bakhtin-inspired metaphor of authorship paves the way for developing the learner’s commitment to voicing opinions as tentative, i.e., as temporary suggestions for claims to knowing in an atmosphere of the recognition of the unfinalizable nature of knowledge. Consequently, developing voiced authoring is not simply about finding and maintaining a unique identity in life, or appropriating surrounding voices for one’s own interest to function in given social practices. Rather, it refers to the critical acts of self-reflexive exploration of personal concerns, interests, problems, or questions in socially responsive contexts, where there is a chance to be heard and respected. As discussed above, in current times—the age of pluralism and globalism—there is a need of learning to critically reflect on multiple, often conflicting, perspectives and worldviews, and to voice own opinions respectfully (cf.Brandist et al., 2017; Teo, 2019).

What, then, could be gained by considering dialogical metaphors for learning in contemporary society, and what distinguishes them from established assumptions in this area? As suggested, the concepts of voice, polyphony and authoring/authorship indicate a radical shift from the dominant focus on reproductive skills dictated ‘from above’ (from society, the state, teachers, and so on) to considering the significance of creative transformation, contingent on multi-voiced dialogic contexts, where new-thinking and originality are actively promoted as part of institutional practices. Authorship, furthermore, implies a performative and communicative view on learning: learning as creatively addressing and responding to others’ unique voices. In order to develop unique and personified voices, we require practices that promote individualized personalization, that means, identity-based development including critical scrutiny of existing voices and culturally dominant discourses, rather than mere adaption to values and norms promoted by authoritarian voices.

## From Socialization to Authoring

In Bakhtin’s parlance, accentuating unilateral socialization generally represents succumbing to a finalizing epistemology, in contrast to recognizing the infinite, open-ended nature of living encounters with others: the unrepeatable uniqueness of ongoing events, experiences, communicative events and acts. Following Bakhtin’s (1990) suggestion, the *once-occurrent* (unrepeatable) nature of events should be seen as an important premise for learning and sharing of experiences as an alternative to viewing knowing as a universal, ideal state of stability already achieved. We therefore suggest learning through recognizing the *once-occurring nature of events* as another figurative concept of value for a dialogic interpretation of learning. Rendering knowing as to some extent provisional and unstable, as genuinely contingent on the other-oriented nature of interactional practices and social circumstances, the openness and creativity in appropriating experiences in social practices is foregrounded.

The recognition of the opportunities to profit from once-occurring events should not be seen as in total conflict with the needs to familiarize people with established forms of knowing. Socialization through education is in many cases a necessary element for maintaining and reproducing important features of existing conventions and cultural and institutional practices. However, as educational scholars recently have emphasized (e.g., Biesta, 2016), it is necessary to be cautious about over-emphasizing the reproductive functions of schooling. One of the significant dilemmas is to support learning trajectories where students recognize the need of cultivating creativity by developing their own values and ideas.

Being socialized is indeed a complex and dynamic process to which individuals most often do not conform blindly. Ultimately, the individual learning processes characterizing traditional patterns of socialization also relies on the learners’ own voice, but here in a weaker sense, since patterns of growth generally are prescribed even when they concern capacities such as learning to collaborate or developing creativity. Thus, learning in the sense of mastering activity-specific skills, and activity-specific cultural tools, does not emphasize the individual voice to the same extent as personalized authoring in the context of other voices does. The “acquisition metaphor”, which refers to the human mind as a container to be filled with conceptual knowledge owned by the individual (Sfard, 1998, p. 6) exemplifies this stress on knowledge as a reified, static, and individual entity and which furthermore privileges the mental level. This stands in stark contrast to the Bakhtin-inspired learning metaphors, which refer to the existential experiences of the learner, and the polyphonic meaning-making at stake in and through complex layers of sociality characterizing socio-historical realities, multi-voiced discourses as well as interpersonal, once-occurring events. Hence, Bakhtin’s contribution to our interpretation of what it means to learn should be seen as a turn to the existential and personal dimensions of learning experiences, as an alternative to focusing on merely cognitive reductionism. Intellectual consciousness is thus always accompanied by evaluative emotions, personal interpretations and implicit or explicit ideological stance-taking (Bakhtin, 1990, 1999).

Adopting a radical dialogic perspective in the spirit of Bakhtin, also leads to critical considerations of the reduction indicative of the metaphor of participation pointed to by Sfard. Even if the learning dimension of encounters is expanded to include the situatedness in social and cultural practices, the emphasis is still heavily on learning as socialization in a setting where authority patterns are given. Learning to move from the periphery to the centre of a community of practice, as the metaphor of participation implies, is yet another form of learning to master the master’s practices. This is not to say that such ways of knowing should be eliminated or undermined. Appropriating skills certainly benefits from a master-apprentice format of learning. However, in a dynamic and changing environment even such settings may profit from the elements added by more radical dialogic theorizing where the authority relationships encourage contributions that go beyond the cultural givens.

Initially, we problematized the implied reductions of positivist approaches to learning: the behaviorist and cognitivist research traditions, which not only reduce the social and dialogic dimensions of learning, but also lead to an emphasis on *reproduction*. In such traditional accounts of socialization, the role of the learner’s voice is largely restricted to adaptation: adaptation to existing values and to socio-historical corpora of accumulated knowledge. Though Dewey’s pragmatism clearly opposed this restricted position, in its recognition of students’ needs, individuals’ life experiences and the intricate complexity between social, cultural, and individual dimensions of knowledge, his conceptual approach to learning and communication seems more instrumentally oriented in comparison to Bakhtin’s existential focus on personalized experiences and expressions. His pragmatist philosophy ultimately acknowledged knowledge building for the sake of usefulness (to society etc.).

We have discussed how the problem of learning is construed in the different perspectives. At one end we find the traditional focus on reproduction where learning is primarily conceived as directed towards preserving established patterns of behaving and thinking. This amounts especially to behaviorism, cognitivism, and pragmatism. At the other end we find sociocultural interpretations that are more or less oriented to learning as a matter of transcending established norms, conventions, cultural tools or existing practices. As the Bakhtinian sociocultural version stresses personalized knowledge processes which point to the potentialities of the unknown that require open-minded new-thinking from all participants involved, it is not idealizing socio-historical traditions and its cultural tools/institutions per se. In that sense, the latter epistemological approach goes beyond a pre-given route of socialization, instead opening for unpredictable knowledge forms that are welcome in our risky, uncertain type of modernity, as discussed. Furthermore, it includes cultivating learning that have to do with polyphonic authoring and with appropriating the experiences of others, without necessarily agreeing or accepting them. Knowing is thus seen as infinite, and as a subject to creative transformation through agentic meaning-making in dialogues characterized by conflictual tensions produced by diverging voices, accents, and the acceptance of ontological plurality. At this level, the Bakhtinian perspective on communication, and its implications for human development, adds important elements to our ways of talking about and theorize learning.

## Conclusions

The basic assumption of our discussion is that theoretical perspectives metaphors of learning emphasize different features of what it means to learn. We have argued that metaphors of learning are important since they foreground certain features of such activities while making others less visible or central. For instance, by localizing learning solely within the individual brain, the collective and socio-material nature of knowing and learning is given less attention. By emphasizing and accepting the authority of established claims to knowledge and singular truths, learning is reduced to a process of subordination to what is already given.

Accordingly, we have foregrounded the tension between reproductive conceptions, which underpin functionalist interpretations of social life and the role of dominant institutional forms of learning, and the alternatives suggested by sociocultural perspectives, especially the dialogical (sociocultural) perspective informed by Bakhtin. The Bakhtinian emphasis on the open-ended nature of living encounters with others, and the expropriation of the intentions of others in communicative events may be seen as a premise for an interpretation of learning as a never-ending feature of human interaction in which we agree, partially agree and disagree on claims to truth. Learning acquires an existential meaning since our experiences are confronted with those of other people. It is moreover important to observe that a dialogical interpretation of learning does not deny the role of socialization, and the appropriation of authoritative discourses by the learner. On the contrary, educational practices in societies have to make citizens familiar with dominant forms of knowing and expression. But, in complex societies learning is intimately interlinked with social responsivity and the continuous engagement in open-ended multi-voiced dialogues. In comparison to Vygotsky’s sociocultural interpretation, Bakhtin makes us considerably more aware of the unpredictable and unfinalized nature of learning in which social encounters offer new ways of seeing and interpreting events; ways that are filtered through the experiences of other people. He also stressed the role of individuals’ agency in dialogic communication. With Bakhtin’s dialogic conceptions, we adhere to a perspective of learning that includes the recognition of individual development beyond the prescribed: an approach to interpreting learning that acknowledges individual agency in its full potential. This builds on an epistemology where knowledge originates in encounters between unique voices that contribute to ongoing and never-ending conversations between partners, willing to learn.

Translated to the field of pedagogy, as reflected in the metaphor “authorial learning”, such a take highlights the notion of personalized knowing, and the legitimate right to express your voice as a learner even when expressing marginal views which do not embrace mainstream discourses (e.g., the voices of authorities). In this context, the metaphorical notion of polyphonic *authoring* makes sense to us as a thought-provoking conceptual alternative to conventional notions of learning. It clearly goes beyond the idea of socialization as the basic skill in life.
